# Supplementary material for: Cerebellar Purkinje Cells Control Posture in Larval Zebrafish (Danio rerio)
Source: bioRxiv. 2024 Nov 9:2023.09.12.557469. Originally published 2023 Sep 14. Preprint. [Version 3] doi: 10.1101/2023.09.12.557469 (PMC10515840; doi:10.1101/2023.09.12.557469)
Supplement: 1 [file NIHPP2023.09.12.557469V3-supplement-1.pdf]

bioRxiv preprint doi: https://doi.org/10.1101/2023.09.12.557469; this version posted November 9, 2024. The copyright holder for this preprint (which was not certified by peer review) is the author/funder, who has granted bioRxiv a license to display the preprint in perpetuity. It is made available under aCC-BY 4.0 International license.

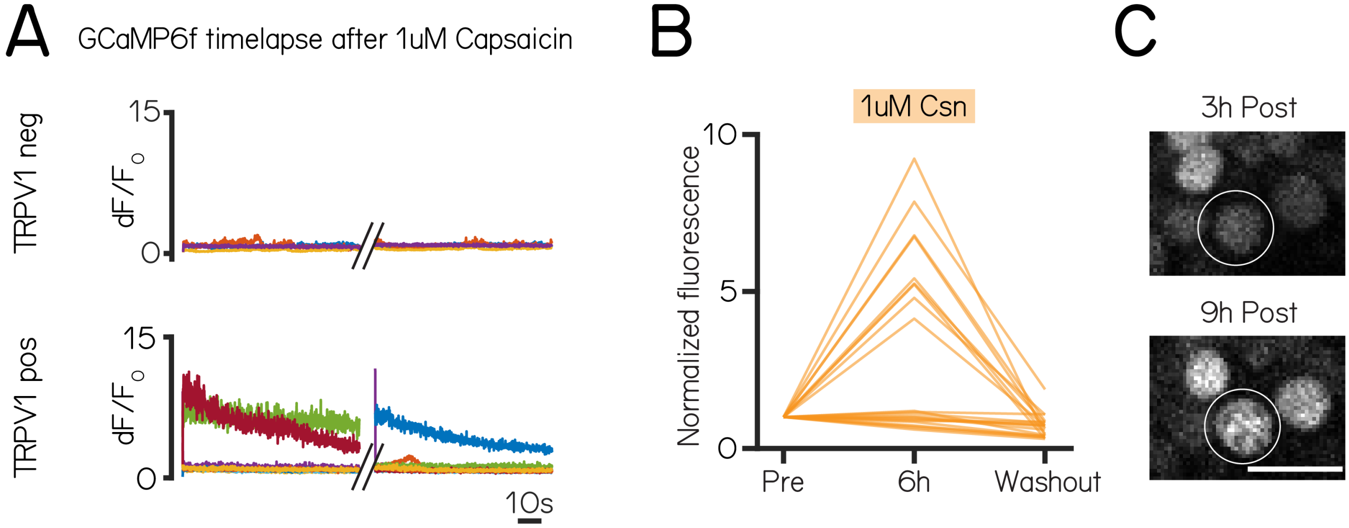

Figure S1: Chemogenetic activation of Purkinje cells is reversible.
(A) Calcium imaging time series after 1 µM Capsaicin of TRPV1-(top) and TRPV1+ cells (bottom) in *Tg(aldoca:TRPV1-tagRFP);Tg(elavl3:h2b-GCaMP6f)* larvae. Two 1.5min time series were recorded showing different cells being active (dF/F0 > 2) at different timepoints (timelapse 1: TRPV1-: 0/16 (0%) cells and TRPV1+: 5/27 (19%) cells activated; timelapse 2: TRPV1-: 0/16 (0%) cells and TRPV1+: 4/27 (15%) cells activated. **(B)** Normalized change in fluorescence following treatment with 1 µM capsaicin at 6h post treatment and after washout in individual Purkinje cells from *Tg(aldoca:TRPV1-tagRFP);Tg(elavl3:h2b-GCaMP6f)* larvae. **(C)** Example confocal image of Purkinje cell nuclei after 3 and 9h of 1 µM capsaicin treatment. Speckled fluorescence could be observed after 9h of 1 µM capsaicin treatment indicative of cell death (white circle). Scale bar 10 µm.

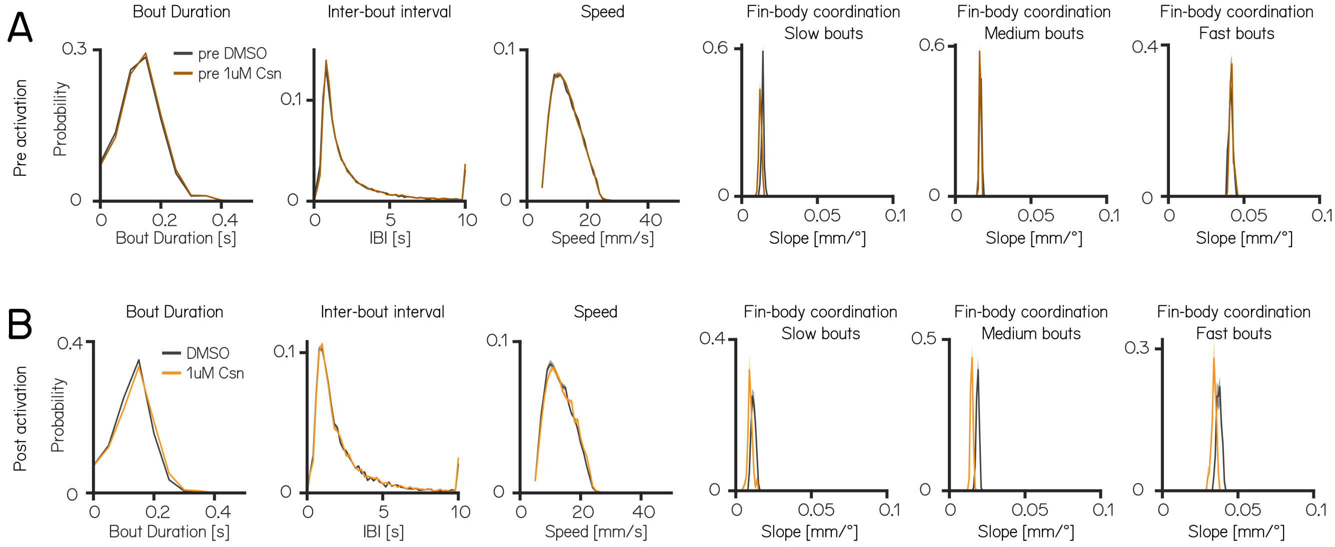

Figure S2: Swim kinematics are not affected by 1 µM capsaicin treatment.
(A) Distributions of swim kinematics and fin body coordination prior to 1 µM capsaicin treatment for control group (pre DMSO - grey) and 1 µM capsaicin group (pre 1 µM capsaicin - brown) reported in table 1. **(B)** Distributions of swim kinematics and fin body coordination during activation for control (DMSO - grey) and 1 µM capsaicin treated (orange) groups reported in table 1.

bioRxiv preprint doi: https://doi.org/10.1101/2023.09.12.557469; this version posted November 9, 2024. The copyright holder for this preprint (which was not certified by peer review) is the author/funder, who has granted bioRxiv a license to display the preprint in perpetuity. It is made available under aCC-BY 4.0 International license.

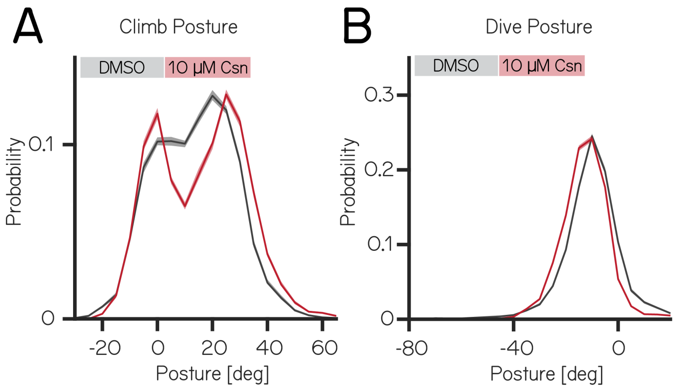

Figure S3: Purkinje cell lesion at 14dpf affects the distribution of postural angles for climb and dive bouts
(A) Probability distribution of climb postures for control (black) and 10 μM capsaicin treated 14dpf larvae (red). Data is shown as median and inter-quartile range. **(B)** Probability distribution of dive postures for control (black) and 10 μM capsaicin treated 14dpf larvae (red). Data is shown as median and inter-quartile range.

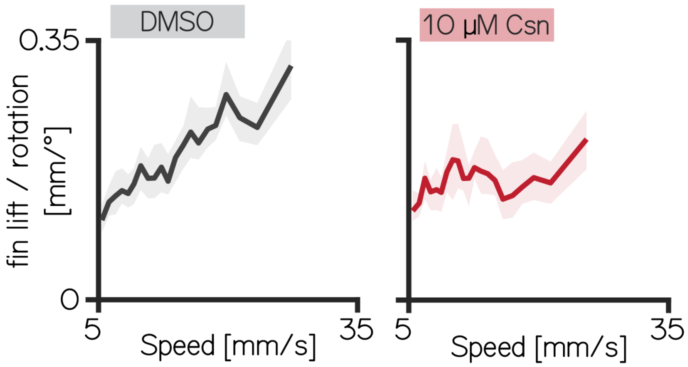

Figure S4: Fin engagement is speed dependent
(A) Fin Lift / rotation ratio versus speed for 14dpf DMSO treated fish (Spearman correlation coefficient: 0.2193). Data is shown as median with 95% confidence interval of the median. **(B)** Fin Lift / rotation ratio versus speed for 14dpf fish 10 μM capsaicin treated fish. Data is shown as median with 95% confidence interval of the median (Spearman correlation coefficient: 0.0397).

bioRxiv preprint doi: https://doi.org/10.1101/2023.09.12.557469; this version posted November 9, 2024. The copyright holder for this preprint (which was not certified by peer review) is the author/funder, who has granted bioRxiv a license to display the preprint in perpetuity. It is made available under aCC-BY 4.0 International license.

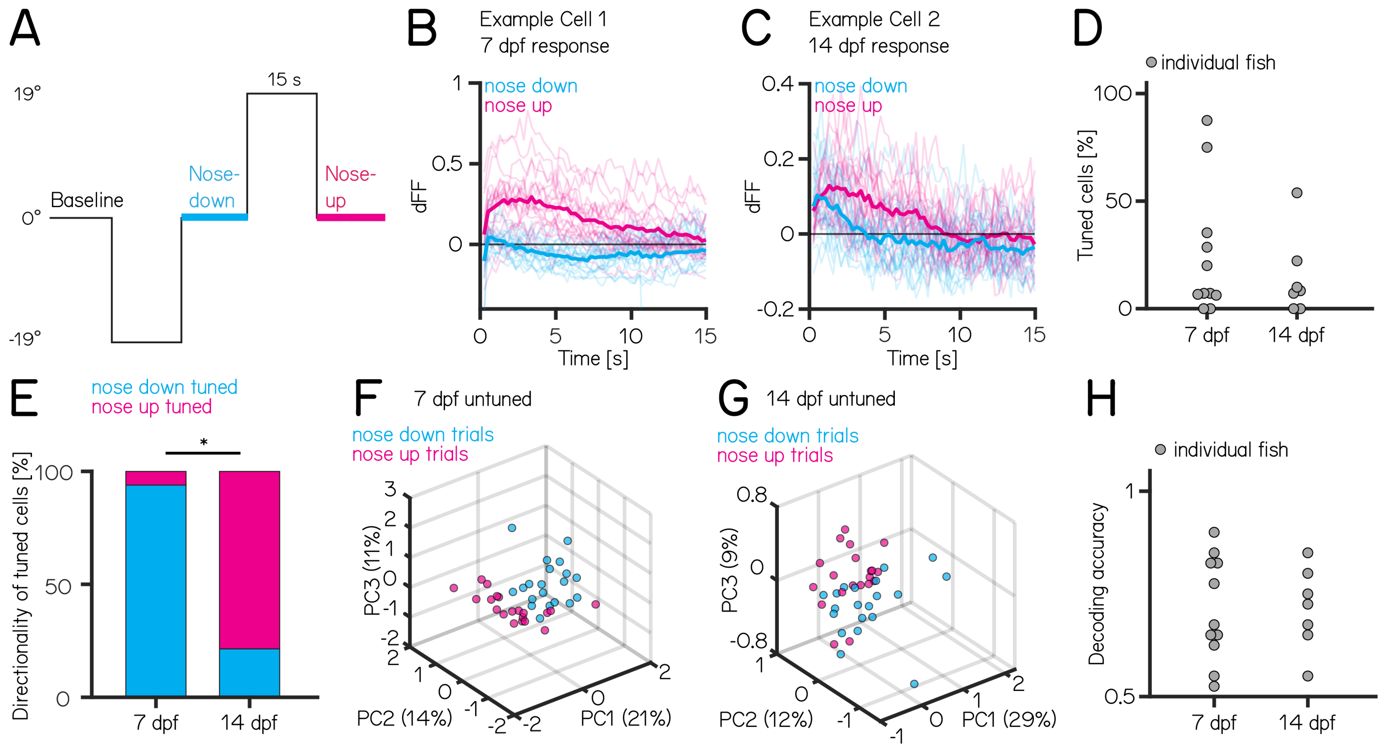

Figure S5: Purkinje cell tuning direction shifts across development, population coding strength remains stable.
(A) One trial consisted of rapid galvanometer steps for 15 seconds in the nose down (-19°, blue) and nose-up (+19°, pink) direction. **(B)** Example responses (n=40) from a single Purkinje cell at 7 dpf to nose-down (blue) and nose-up (pink) pitch tilts. The thicker lines indicate the median response to all nose-down or nose-up trials. **(C)** Example responses (n=40) from a single Purkinje cell at 14 dpf to nose-down (blue) and nose-up (pink) pitch tilts. The thicker lines indicate the median response to all nose-down or nose-up trials. **(D)** Percentage of tuned cells from individual fish based on a directionality index larger than ±0.35 (median [inter-quartile range]:7 dpf: 7 [6 – 34]%; 14 dpf: 8 [2 – 19]%; p-value = 0.7763, Wilcoxon rank sum test). **(E)** Direction of tuned cells at 7 and 14 dpf (7 dpf: 2/31 cells up/down-tuned; 14 dpf 11/3 cells up/down-tuned, p-value < 0.001, Fisher's exact test). **(F)** Principal component analysis of all untuned cells at 7 dpf for each of 20 up (pink) and 20 down (blue) trials. (Percentage of variance explained) **(G)** Principal component analysis of all untuned cells at 14 dpf for each of 20 up (pink) and 20 down (blue) trials. (Percentage of variance explained) **(H)** Performance of a support vector machine for binary classification of up/down tilt using the responses from untuned neurons. Dots are individual fish at 7 dpf and 14 dpf (median [inter-quartile range]: 7 dpf: 0.68 [0.63 – 0.83]; 14 dpf: 0.73 [0.65 – 0.79]; p-value = 0.9468, Wilcoxon rank sum test).
