# Supplementary material for: Cerebellar Purkinje Cells Control Posture in Larval Zebrafish (Danio rerio)
Source: bioRxiv. 2024 Mar 5:2023.09.12.557469. Originally published 2023 Sep 14. Preprint. [Version 2] doi: 10.1101/2023.09.12.557469 (PMC10515840; doi:10.1101/2023.09.12.557469)
Supplement: Supplementary file 1 [file NIHPP2023.09.12.557469v2-supplement-1.pdf]

bioRxiv preprint doi: https://doi.org/10.1101/2023.09.12.557469; this version posted March 5, 2024. The copyright holder for this preprint (which was not certified by peer review) is the author/funder, who has granted bioRxiv a license to display the preprint in perpetuity. It is made available under aCC-BY 4.0 International license.

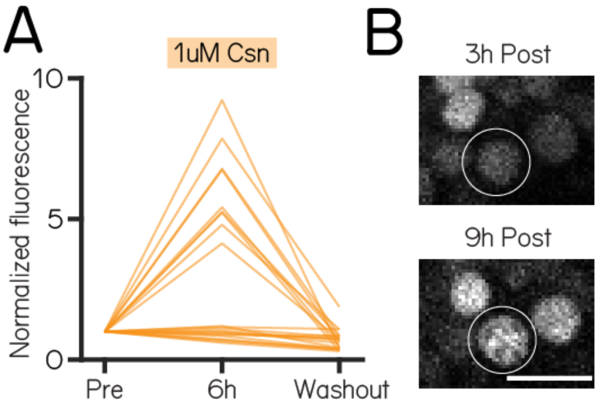

Figure S1: Chemogenetic activation of Purkinje cells is reversible.
(A) Normalized change in fluorescence following treatment with 1 μM capsaicin at 6h post treatment and after washout in individual Purkinje cells from *Tg(aldoca:TRPV1-tagRFP);Tg(elavl3:h2b-GCaMP6f)* larvae. **(B)** Example confocal image of Purkinje cell nuclei after 3 and 9h of 1 μM capsaicin treatment. Speckled fluorescence could be observed after 9h of 1 μM capsaicin treatment indicative of cell death (white circle). Scale bar 10 μm.

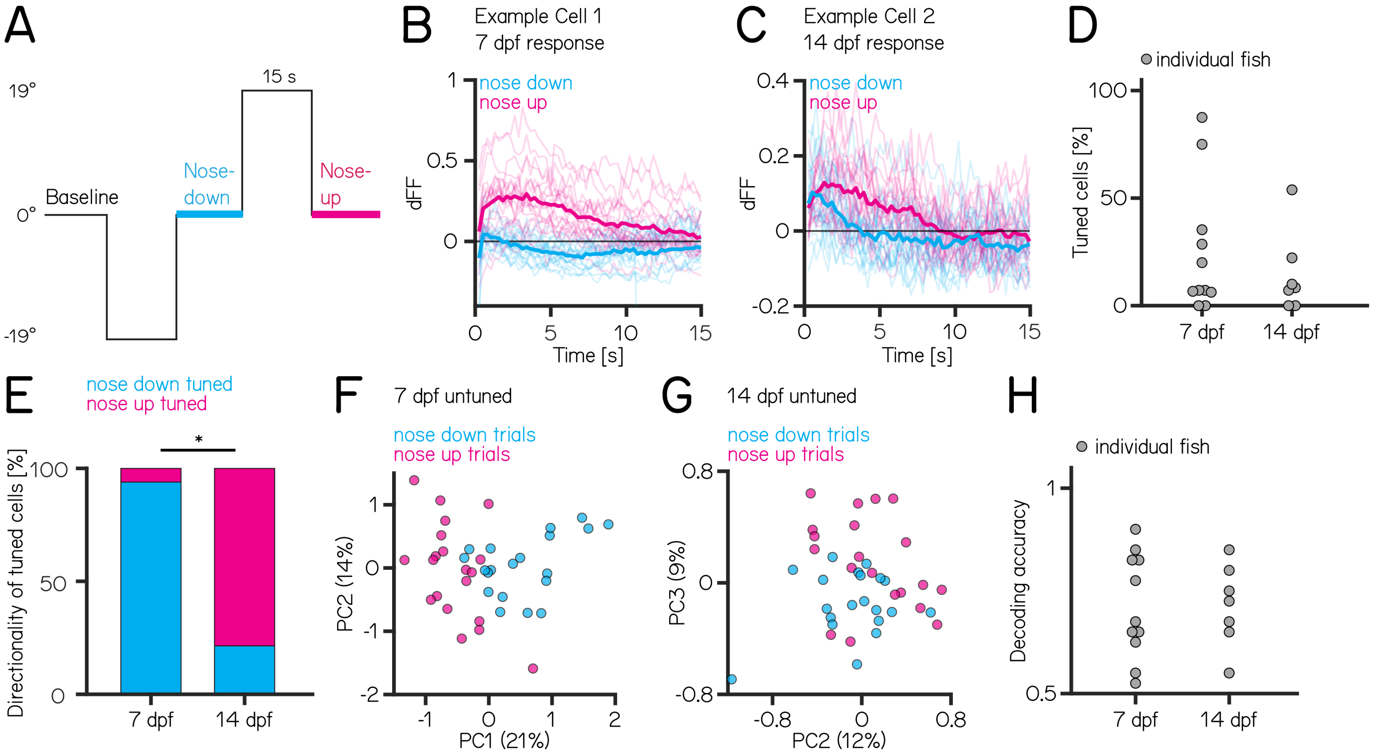

Figure S2: Purkinje cell tuning direction shifts across development, population coding strength remains stable.
(A) One trial consisted of rapid galvanometer steps for 15 seconds in the nose down (-19°, blue) and nose-up (+19°, pink) direction. **(B)** Example responses (n=40) from a single Purkinje cell at 7 dpf to nose-down (blue) and nose-up (pink) pitch tilts. The thicker lines indicate the median response to all nose-down or nose-up trials. **(C)** Example responses (n=40) from a single Purkinje cell at 14 dpf to nose-down (blue) and nose-up (pink) pitch tilts. The thicker lines indicate the median response to all nose-down or nose-up trials. **(D)** Percentage of tuned cells from individual fish based on a directionality index larger than ±0.35 (median (interquartile range):7 dpf: 7 (6-34)%; 14 dpf: 8 (2-19)%; p-value = 0.7763, Wilcoxon rank sum test). **(E)** Direction of tuned cells at 7 and 14 dpf (7 dpf: 2/31 cells up/down-tuned; 14 dpf 11/3 cells up/down-tuned, p-value < 0.001, Fisher's exact test). **(F)** Principal component analysis of all untuned cells at 7 dpf for each of 20 up (pink) and 20 down (blue) trials. (Percentage of variance explained) **(G)** Principal component analysis of all untuned cells at 14 dpf for each of 20 up (pink) and 20 down (blue) trials. (Percentage of variance explained) **(H)** Performance of a support vector machine for binary classification of up/down tilt using the responses from untuned neurons. Dots are individual fish at 7 dpf and 14 dpf (median (interquartile range): 7 dpf: 0.68 (0.63-0.83); 14 dpf: 0.73 (0.65-0.79); p-value = 0.9468, Wilcoxon rank sum test).
