# Supplementary material for: Purkinje Cells Control Posture in Larval Zebrafish (Danio rerio)
Source: bioRxiv. 2023 Sep 14:2023.09.12.557469. Preprint. [Version 1] doi: 10.1101/2023.09.12.557469 (PMC10515840; doi:10.1101/2023.09.12.557469)
Supplement: Supplementary file 1 [file NIHPP2023.09.12.557469v1-supplement-1.pdf]

bioRxiv preprint doi: https://doi.org/10.1101/2023.09.12.557469; this version posted September 14, 2023. The copyright holder for this preprint (which was not certified by peer review) is the author/funder, who has granted bioRxiv a license to display the preprint in perpetuity. It is made available under aCC-BY 4.0 International license.

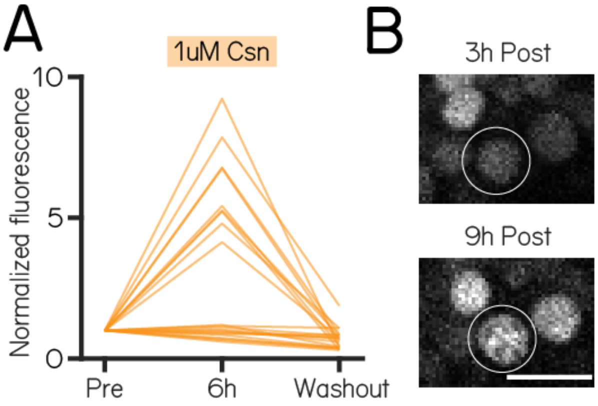

Figure S1: Supplemental figures for 1 μM capsaicin experiments.
(A) Normalized change in fluorescence following treatment with 1 μM capsaicin at 6h post treatment and after washout in individual Purkinje cells from *Tg(aldoca:TRPV1-tagRFP)*;*Tg(elavl3:h2b-GCaMP6f)* larvae.
(B) Example confocal image of Purkinje cell nuclei after 3 and 9h of 1 μM capsaicin treatment. Speckled fluorescence could be observed after 9h of 1 μM capsaicin treatment indicative of cell death (white circle). Scale bar 10 μm.
